# Examining the concept of *One Health* for indigenous communities: A systematic review

**DOI:** 10.1016/j.onehlt.2021.100248

**Published:** 2021-04-14

**Authors:** Sean A. Hillier, Abdul Taleb, Elias Chaccour, Cécile Aenishaenslin

**Affiliations:** a School of Health Policy & Management, Faculty of Health, York University, 348A Stong College, 4700 Keele St., Toronto, Ont M3J 1P3, Canada; b Dalla Lana School of Public Health, University of Toronto, Canada; c Health Policy & Equity, School of Health Policy & Management, Faculty of Health, York University, Canada; d Département de pathologie et microbiologie, Faculté de médecine vétérinaire, Université de Montréal, Canada

**Keywords:** One health, Indigenous health, Indigenous, First nations, Inuit, AMR

## Abstract

**Purpose:**

This paper examines whether the usage of the concept of *One Health* in Canada-based research aligns with traditional Indigenous notions of health and wellness.

**Methods:**

A comprehensive search of the literature was conducted using primary databases, including Scholars Portal, ProQuest Social Science, Sociological Abstracts (ProQuest), OVID Healthstar, Embase, Medline, Pubmed and Google Scholar. Papers discussing *One Health* and Indigenous Health were selected and analyzed through Nvivo12 to generate common themes across the studies.

**Results:**

The analysis identified three major themes that focused on *One Health* as it relates to climate change, zoonosis, and social relationships between humans and animals. Climate change was seen to have affected the environmental health of Northern latitude areas where many Indigenous communities reside. Infectious diseases within Indigenous communities were a frequent topic of study and indicated that infections transmitted by dogs are likely to be addressed with *One Health* interventions. *One Health* interventions are likely to equally address the health of humans, animals, and the environment.

**Conclusions:**

No significant connection between *One Health* and Indigenous knowledges was established in the analyzed articles. Articles discussed *One Health* as it pertains to epidemiological surveillance and research. The implications of utilizing *One Health* towards Indigenous Peoples and culture were not explicitly addressed.

## 1 Introduction

Indigenous Peoples across Canada are unified in their belief that the wellbeing of an individual is directly connected to the wellbeing of the land. Across modern-day Canada, Indigenous Peoples have lived in harmony with the land since time immemorial. However, with the arrival of colonial settlers and the forceful removal of autonomous, prospering nations from their traditional territories, Indigenous ways of living, being, and knowing were forever altered [1]. Colonisation and the actions of European settlers continue to affect the health outcomes of Indigenous Peoples today, with modern-day policies such as the Indian Act (1874, amended in 1985) propagating notable health inequities [2,3]. Nonetheless, Indigenous Peoples have demonstrated resilience and survival in the face of systemic targeting by past and present colonial policy [4].

Western conceptualizations primarily define physical health through a deficit-based approach, as the absence of illness and disease [5]. Alternatively, Indigenous Peoples and communities in Canada and around the world, take a holistic approach, whereby wellness is defined as a balance between physical, emotional, mental, and spiritual health [6]. The Medicine Wheel, which presents health as an equal balance between the physical, emotional, spiritual and mental aspects of human beings, is a prime example of this holistic First Nations specific understanding of health [5]. The perseveration of the land, by extension, is inextricable from the overall health of humans (i.e., stewards of said land) [7].

In recent years, Western-based epistemologists have made significant strides to incorporate holistic concepts, including stewardship of the land, in their conceptualization of health. The World Health Organization (WHO) cites ‘stewardship’ as a critical function of a health system [7]. A prime example of this concept is the notion of *One Health*. As articulated by the WHO in 2017, *One Health* is defined as the use of multidisciplinary approaches in the implementation of policy design and public health interventions. It recognizes that the health of humans is intricately tied to the health of animals and the environment [8]. Stakeholders from interdisciplinary sectors such as clinical health, public health, environmental science, veterinary science, plant health, political science, and the social sciences are encouraged to collaborate in developing interventions that best consider the combined health outcomes of all the intertwined elements [9]. However, to date, there has been little emphasis placed on understanding and incorporating traditional Indigenous knowledges within the *One Health* framework. This paper will answer two research questions: First, what are the main issues/topics addressed by One Health research conducted with Indigenous communities? And second, how do researchers engage with Indigenous communities and reflect on Indigenous knowledges while conducting research informed by *One Health*?

## 2 Methods

A comprehensive search of the literature was conducted in January 2020 and updated in November 2020. The following electronic databases were used to scan the relevant literature: Scholars Portal, ProQuest Social Science, Sociological Abstracts (ProQuest), OVID Healthstar, Embase, Medline, Pubmed and Google Scholar. A single full-text search strategy was adopted, using the following keywords across the databases: (‘One Health’ OR ‘OneHealth’) AND (‘Indigenous’ OR ‘Aboriginal’ OR ‘Métis’ OR ‘First Nations’ OR ‘Inuit’). The term ‘Indigenous’ is used throughout this review to broadly refer to this ethnic group of people as it the most inclusive title internationally (legally, the term ‘native’ has no status in Canada). While key terms provide a reference to Indigenous groups in Canada. International studies from other settler-colonial countries, including the United States and Australia, were included, as they provide complementary information relevant to the topic of this study.

In order to be included, studies had to:

• be published in English;
• be published after January 2000;
• mention *One Health* in the full text;
• contain findings with implications related to Indigenous Peoples.

Studies were further excluded if they:

• mentioned *One Health* as an institution as opposed to a concept or a framework;
• largely pertained to the general population (i.e. Indigenous Peoples were not the focus, despite possibly being mentioned);
• used the term “Indigenous” in a microbiological concept to describe cells and other species;
• were symposium reviews.

Screening of titles and abstracts was conducted independently by two analysts. Duplication and studies that did not meet all inclusion and exclusion criteria were removed. NVivo 12 was used for detailed analysis of full texts from each study and to generate common themes across studies. The articles were further organized based on the type of study, region in which the study is conducted, and the specific *One Health* research area explored in the article.

In total, the search yielded over 747 results from all databases, from which 727 studies were deemed outside of the scope and excluded. The remaining 20 studies met all inclusion criteria. A complete list of these articles is presented in Table 1.

**Table 1:** Summary of Literature

Reference⁎	Region	Type of study or methods	*One Health* area of focus
[14] Aenishaenslin et al. (2014)	Nunavik	Epidemiological Study	Zoonosis /Rabies
[26] Bailey et al.(2018)	N/A	Review	Zoonosis
[28] Baker et al. (2020)	Arctic	Epidemiological Study	Animal-Assisted Interventions
[15] Beknazarova et al. (2019)	Northern Australia	Epidemiological Study	Zoonosis
[19] Beknazarova et al. (2020)	Northern & Central Australia	Epidemiological Study	Zoonosis
[17] Brookes et al. (2017)	Northern Australia	Qualitative (Interviews)	Zoonosis/Rabies
[18] Brookes et al. (2020)	Northern Australia	Qualitative (Interviews)	Zoonosis
[27] Chalmers & Dell, (2015)	N/A	Forum/Review	Animal-Assisted Interventions
[24] Degeling et al. (2018)	Northern Australia	Qualitative (Interviews)	Zoonosis/Rabies
[10] Dudley et al. (2015)	Arctic	Review	Climate Change & Zoonosis
[23] Smout et al. (2017)	Northern Australia	Epidemiological Study	Zoonosis
[11] Hueffer et al. (2019)	Arctic	Review	Climate Change
[9] Mazet et al. (2014)	N/A	Review	Zoonosis
[13] Parkinson et al. (2014)	Arctic	Forum/Review	Climate Change
[16] Riley et al. (2020)	Northern Australia	Epidemiological Study	Zoonosis
[25] Rock et al. (2017)	Alberta	Ethnographic Case-Study	Zoonosis/Rabies
[12] Ruscio et al. (2015)	Arctic	Review	Climate Change
[20] Schurer et al. (2013)	Saskatchewan	Epidemiological Study	Zoonosis
[21] Schurer et al. (2014)	Saskatchewan	Epidemiological Study	Zoonosis
[22] Schurer et al. (2015)	Saskatchewan	Epidemiological Study	Zoonosis/Rabies

⁎ This chart lists each article and indicates the region where the studied occurred, the type of study, and the area(s) of *One Health* discussed in the articles.

## 3 Results

### 3.1 *One Health* research conducted with Indigenous communities

Three major research themes intersecting *One Health* and Indigenous health were identified from our analysis (Table 1):

1. Climate Change
2. Zoonosis
3. Social Relationships between Humans and Animals

An introduction into the scope of those themes from our analysis of the articles is shown below.

#### 3.1.1 Climate change

Four articles analyzed the health impacts of climate change on Northern residents near the Arctic Circle [[10], [11], [12], [13]]. The rate and impact of climate change is faster in northern latitudes than in more temperate areas, having detrimental effects on northern Indigenous communities [10]. Warming Arctic temperatures have disrupted the migration patterns of animals, thus hindering the ability of Indigenous Peoples across the north to hunt their traditional game with the same predictability as before [11]. Climate change has also increased the likelihood of zoonoses due to more significant opportunities for pathogens to spread in warmer temperatures [10]. Dudley et al. [10], as well as Ruscio et al. [12], discuss how a changing Arctic environment has exacerbated the spread of rabies and other parasites due to pollution and global warming. Warming climates also have a disruptive effect on the essential cultural practices of northern Peoples, which include tracking and hunting animals, as well as seasonal migrations relying on thick frozen sea ice [11,13]. *One Health* was described as a potentially useful framework for interventions addressing climate change-induced health problems.

#### 3.1.2 Zoonosis

Zoonosis was an area of focus in fifteen of the 20 articles analyzed. The studies focused on infections that occur as a result of close interactions between humans and animals. The most significant of the zoonotic infections discussed were rabies and helminths, for which dogs are a vector [[14], [15], [16]]. Free-roaming dogs are numerous in Indigenous communities throughout northern Saskatchewan, Nunavik, and Northern & Central Australia. Nine articles described the state of dog borne infectious diseases within those regions through epidemiological surveillance of dog bites and feces [14,15,[17], [18], [19], [20], [21], [22], [23]]. The articles highlight the vaccination of dogs and post-exposure prophylaxis (PEP) as interventions resulting in positive health outcomes for both humans and animals, in alignment with the *One Health* paradigm [14,[20], [21], [22],^24^,^25^]. Community concerns over public health interventions imposed by cities or Western health agencies were voiced in three articles [10,22,24]. *One Health* could address these concerns via interdisciplinary collaboration and consultation during the development of public health interventions [9,26].

#### 3.1.3 Social relationships between humans and animals

*One Health* interventions aim to improve both animal and human health. Seven articles described the vital relationship that Indigenous communities have with animals [14,22,[24], [25], [26], [27], [28]]. From the context of *One Health*, it is important to consider that infectious diseases such as rabies are spread through interactions with said animals. Therefore, the health of both humans and animals is intertwined. Six of the articles mentioned how performing culturally unsafe interventions on dogs such as mass culling or body alterations could harm Indigenous communities if no prior consultation occurs [9,14,21,22,24,25]. The connection between communities and the animals that live within them was discussed as a positive promoter of health [22,24,25]. Though, when animals become vectors for infection, a beneficial *One Health* intervention must impact the health of animals, as opposed to just minimizing the risk to humans [14,24,25].

### 3.2 Engaging with Indigenous communities

Four major research themes emerged relating to how researchers used a One Health framework to engage with Indigenous communities. This includes how researchers may have reflected upon or integrated Indigenous knowledges and ways of knowing or being into the work they were conducting. The four themes presented are:

1. *One Health* as a Tool to Study the Health Effects of Climate Change
2. *One Health* as Tool for Reducing the Burden of Zoonoses
3. *One Health* as an Approach to Disease Surveillance
4. *One Health* as an Approach to study Indigenous Concepts of Animals

#### 3.2.1 *One Health* as a tool to study the health effects of climate change

Hueffer et al. [11] conducted workshops with Arctic community stakeholders and residents, resulting in a better understanding of how *One Health* conceptual interventions can be implemented. The discussions identified: improved veterinary care, youth educational programs, improved natural resource management, improved surveillance of issues around food security, and better understanding the sustainability of resources to address concerns regarding the changing ecological health of the Arctic [11]. Community consultation with the stakeholders was necessary to identify which issues are the priority of residents, and how best they can be addressed. The authors considered the *One Health* approach to be a holistic framework for population-level intervention that best improves the wellbeing of animals, humans, and the environment [11].

With the increasing urbanization of the earth's population, it is more difficult to witness the effects of human activities on areas outside of the major urban spaces. This lack of insight is exceptionally accurate for the circumpolar region and residents (substantially made up of Indigenous Peoples) of northern latitudes whose livelihoods continue to be impacted by climate change. Dudely et al. [10] argue that policies that address climate change in the Arctic are best applied with a *One Health* approach that engages ecological and environmental sciences. Indigenous inhabitants of northern latitudes have historically relied on hunting and gathering as means of sustenance [11]. Climate change is detrimental to the Peoples' ability to hunt and gather, as the environment becomes less predictable and harder to navigate by humans [12]. Non-holistic interventions aimed at addressing the resulting food insecurity intercede at the downstream determinants (e.g., easier access to less-nutritious, non-traditional foods), while ignoring the causal role of climate change [10]. These issues would be best addressed through a *One Health* approach, which prioritizes traditional knowledges through community consultation.

#### 3.2.2 *One Health* as tool for reducing the burden of zoonoses

Zoonotic infections occur when pathogens such as bacteria or viruses, which reside in an animal host, known as a *reservoir host*, infect humans or vice versa [28]. The likelihood of these infections increases with a higher frequency of interaction between animals and humans. For communities that rely on animals for hunting and transport, zoonotic infections pose a public health risk, further exacerbated by climate change [10]. Changing temperatures can also disturb the distribution and diversity of reservoir hosts, increase the frequency of contact between humans and reservoir hosts and increase the probability of cross-species jump [10,28]. As the region around the Arctic Circle warms up faster than any other region, its Indigenous inhabitants are likely to be at the forefront of possible zoonotic infections [10].

Public health concerns for inhabitants in and around the Arctic Circle are compounded by the fact that effective veterinary or animal surveillance methods are lacking [10]. Ruscio et al. [12] concluded that *One Health* approaches to addressing these issues should include tracking the spread of wild pathogens, their hosts, and changes in tropism and susceptibilities. Concerns surrounding ecological and Indigenous health in the Arctic are best addressed through interventions that consider the interconnectedness of climate change, animal behaviour, animal physiology, and zoonosis. As stewards of the land, northern Indigenous communities have witnessed the rapid decline of Arctic ecological health firsthand [7]. Effective *One Health* intervention in the Arctic requires international collaboration between various nations and community consultation with stakeholders that include the Inuit People [12].

#### 3.2.3 *One Health* as an approach to disease surveillance

One specific pathogen endemic to some northern latitudes where Indigenous communities reside is *rabies lyssavirus* (rabies). The health concerns of rabies are best addressed with *One Health* interventions that take into account the diverse species which are susceptible to the virus, which include dogs, cats, bats, and foxes, among others [14]. *One Health* promote epidemiological surveillance on the host reservoirs of the virus and the development of targeted interventions for animals (eg. rabies vaccination program) and humans (eg. dog bite preventive programs) [14]. *One Health* interventions that address the high prevalence of parasites among the roaming dogs in Indigenous communities include better surveillance of said parasites both in animals and in humans [15,19,21]. Such a process was done by Schurer et al. [21] in Southern Saskatchewan Saulteaux First Nations communities, where blood samples were collected from community participants, and blood and fecal samples were collected from local dogs.

#### 3.2.4 *One Health* as an approach to study indigenous concepts of animals

As of 2018, there were no cases of canine-rabies in Australia [24]. However, the spread of the virus within the islands of Southeast Asia means that it could soon be introduced into Australia. If rabies were to become endemic in Northern Australia, there would be implications for Indigenous communities, as well as the dogs and dingoes that live there [24]. Indigenous communities in Northern Australia expressed, through interviews, their fear that public health interventions to combat rabies will be focused on culling or depopulation [24]. This fear by local Indigenous communities stems from a history of distrust between them and governments in Australia [24]. A *One Health* approach to addressing the public health concern must take into account the desires and goals of the local communities, which see to it that dogs and dingoes receive consideration as human wellbeing receives [24]. Culturally safe interventions using the *One Health* concept must be based on community consultation. They can include monitoring, vaccination of dogs, administering PEP, and measures to lessen the frequency of dog bites [24].

Indigenous communities and nations have long acknowledged the interdependence of animals, humans, and the environment [27]. Indigenous Peoples are aware of the benefit that environmental health and human health play for each other [9,27]. In Calgary, Canada, *One Health* approaches can address rabies resulting from dog bites, especially in children, by implementing interventions that minimize the stress experienced by the animal. These include humane sheltering and rehoming of impounded dogs, and subsidized spaying and neutering operations with the consent of the owner or community [25]. Government policies impacting Indigenous Peoples often consider the presence of dogs as a health concern, as depicted by the construction of a school in the Northwest Territories in the 1960s, which was delayed and built 13 km away from a First Nations community due to the incidence of free-roaming dogs within the community [25]. This change had a subsequent effect on the ability of children to access education. A *One Health* approach would require consultation between all vested stakeholders to reach a holistic solution for animals, humans, and environmental health.

In most cases, the mass culling of dogs to protect from rabies and parasites is likely to be met with opposition. Depopulation interventions are not in alignment or harmony with many Indigenous ways of knowing. Contrarily, the Canine Action Plan (CAP) in Saskatchewan used *One Health* methodologies to understand the epidemiology of dog bites and resulting disease, consult with communities, and offer culturally safe interventions [22]. The research process involved collaboration between rural and Indigenous stakeholders, where Elders and community leaders could share their input freely [22]. The result is parasite analysis and serology conducted through veterinary services that are deemed culturally appropriate.

## 4 Discussion

The *One Health* concept graduates from traditional Western definitions of health where health is viewed as the absence of disease rather than holistically [11]. *One Health* approaches include mental and emotional health and examine how they are affected by determinants, such as animal and environmental health [11]. For some Inuit youth, wellness is affected by the reliability of sea ice in the areas surrounding their homes [11]. Inuit and other northern Indigenous Peoples' health are intricately tied to their ability to live on and interact with the land in a manner practiced by their specific cultures and traditions. Climate change and colonisation act to impede the ability of Indigenous Peoples to interact with their environments in the way that they see necessary to maintain and enhance their mental and emotional wellbeing [11]. The rapidly changing environmental factors in the Arctic necessitate a holistic and interdisciplinary approach to address the resulting health concerns for Indigenous Peoples, biota, and the environment. Additionally, there are calls for countries like Australia to embrace a *One Health* approach when addressing public health concern that consider the desires and goals of the local communities [24]. In the articles included in this analysis, *One Health* is presented as a tool to address these challenges.

### 4.1 Missing elements: Traditional knowledge as legitimate knowledge

Throughout our review, articles implied that *One Health* interventions would effectively address the health concerns that impact Indigenous communities. Some of the necessary elements in the design and implementation of interventions include community consultation and multidisciplinary collaboration [12]. When considering the major emerging theme of minimizing rabies infections, the promotion of animal health was discussed as one of the primary *One Health* interventions [14,21,22,25]. However, our analysis did not conclude that these promoted interventions would follow an Indigenous perspective nor consider a particular community's ways of knowing and being. While the interventions were considered by authors to be culturally safe, their intended results minimized potential conflict with the community by compromising a Western methodology, such as dog population control program, rather than embracing an Indigenous perspective of health or holistic wellbeing. There was no indication of any particular intervention incorporating specific Indigenous perspectives or teachings. Most studies cited community consultation as one of their key priorities, but the extent to which input from Indigenous communities was employed was not articulated or demonstrated.

Of the studies that were *One Health* interventions that targeted northern latitudes and Arctic communities noted no specific Indigenous or Inuit teachings being incorporated into their work following the community consultation step in the design of their intervention. Northern Indigenous communities were accurately recognized as the critical stakeholders within Arctic health [[10], [11], [12],^14^]; however, their input in the intervention design was used as a tool for securing community buy-in rather than an explicit guide for the intervention development?

The evaluation processes of the interventions were also not detailed in any of the articles. There was no significant discussion around the implications for Indigenous culture as a result of the threat of climate change or increased prevalence of zoonosis [9,14,15,21,22,24]. The epidemiological studies provided a quantitative description of the state of zoonosis within specific Indigenous communities. The results of the search indicate that *One Health* interventions should be applied when working in collaboration with Indigenous Peoples because *One Health*'s strong correlation with established Indigenous knowledges and practices.

Concepts that could intersect Indigenous knowledges and *One Health* remained mostly missing in included studies, such as discussions regarding the state of vegetation and plant health used for traditional medicines resulting from climate change and its impact on Indigenous food systems and the northern ecosystem. Other missing concepts include any discussion on the specific implications of *One Health* for urban Indigenous individuals and how it could be extended to include the health of Indigenous Peoples not living within a specific Indigenous community.

Finally, *One Health* methodologies are favoured by authors due to their holistic tendencies, which mimic traditional ways of knowing rather than being wholly guided and informed by Indigenous practices. As such, *One Health* is portrayed as a framework that can utilize existing Indigenous traditions to promote a fundamentally positivist interpretation of health. Studies included in this review utilize the concept of One Health to support a holistic rhetoric, but they ultimately apply methodological approaches drawn from Western scientific methods which are anchored in a rather narrow vision of health. Understanding how traditional Indigenous knowedges could be merged to complement and enhance the foundational concepts within *One Health* would strengthen this approach.

One Health and the scholars that use it have the distinct opportunity to take a concept already being used within Indigenous communities and to blend it with Indigenous perspectives, knowledges, and ways of knowing and being to create something truly unique. In the past decade we have seen Two-eyed seeing, or *Etuaptmumk,*being used as a guiding principle of Mi'kmaq knowledge. Bartlett, Marshall, Marshall, & Iwama note: “Two-Eyed Seeing adamantly, respectfully, and passionately asks that we bring together our different ways of knowing to motivate people, Aboriginal and non-Aboriginal alike” [31] (p. 21). Within this framework, Indigenous knowledges and western sciences can interact, allowing for a more diversified understanding of the world [31]. Similarly, but distinctive in their approach, there are guides and frameworks for working with Inuit communities, including the *National Inuit Strategy on Research* which states “Advancing Inuit governance in research is imperative for enhancing the efficacy, impact, and usefulness of research for Inuit. This requires […] partners with Inuit representational organizations to implement engagement processes that respect the role of Inuit in decision-making when it comes to research involving our people, wildlife, and environment.” [32] (p. 4). There is an urgent need to envision a distinctive yet collaborative and respectful *One Health* approach that happens alongside with Indigenous Peoples and organizations.

### 4.2 Study limitations

It is important to note, that there are existing studies which consider and use a *One Health* approach while incorporating Indigenous perspectives of health and wellbeing [29,30]. However, these studies do not describe their approach as ‘*One Health*’, and therefore were not included in this review. Therefore, it is even more important that we develop a comprehensive understanding that ties together Indigenous knowledges and concepts of health and wellbeing with a *One Health* approach. In doing this, we will be better positioned to understand and respond to the unique needs of Indigenous Peoples and communities.

## 5 Conclusion

This review analyzed 20 articles that covered diverse topics within the field of *One Health* and Indigenous health. Of the eleven analyzed articles, three major themes were identified, focusing on the *One Health* implications of climate change, zoonosis, and the social relationships between humans and animals. Climate change was seen to have affected the environmental health of Northern latitude areas where many Indigenous communities reside. Warming climates impact the ability of communities to live and hunt, thereby affecting their health outcomes. Infectious diseases within Indigenous communities were analyzed by many of the articles, and the studies indicate that dog borne infections are likely to be addressed with *One Health* interventions. Some Indigenous communities consider animals to be an equal part of society and would benefit from seeing the health of those animals improved. *One Health* interventions are likely to equally address the health of humans, animals, and the environment. Although Indigenous communities could benefit from interventions that would avoid harmful practices such as the mass culling of dogs, no clear and specific connection was demonstrated between *One Health* and Indigenous ways of knowing. No significant discussion was identified regarding the implications of utilizing the *One Health* on Indigenous Peoples.

## Informed consent

For this type of study formal consent is not required.

## Funding

This study was supported by a grant from the 10.13039/501100000024Canadian Institutes of Health Research (CIHR), Funding Reference No. NGR–167542, with additional research funding from 10.13039/501100000024CIHR Funding Reference No: 43980.

## Declaration of competing interest

The authors declare that they have no conflict of interest.
